# Quantifying similarity of pore-geometry in nanoporous materials

**DOI:** 10.1038/ncomms15396

**Published:** 2017-05-23

**Authors:** Yongjin Lee, Senja D. Barthel, Paweł Dłotko, S. Mohamad Moosavi, Kathryn Hess, Berend Smit

**Affiliations:** 1 Institut des Sciences et Ingénierie Chimiques, Valais, Ecole Polytechnique Fédérale de Lausanne (EPFL), Rue de l'Industrie 17, CH-1951 Sion, Switzerland; 2 Department of Chemical and Biomolecular Engineering, University of California, Berkeley, California 94720, USA; 3 DataShape Group, Inria Saclay Ile-de-France, 91120 Palaiseau, France; 4 SV BMI UPHESS, Ecole Polytechnique Fédérale de Lausanne, CH-1015 Lausanne, Switzerland

## Abstract

In most applications of nanoporous materials the pore structure is as important as the chemical composition as a determinant of performance. For example, one can alter performance in applications like carbon capture or methane storage by orders of magnitude by only modifying the pore structure. For these applications it is therefore important to identify the optimal pore geometry and use this information to find similar materials. However, the mathematical language and tools to identify materials with similar pore structures, but different composition, has been lacking. We develop a pore recognition approach to quantify similarity of pore structures and classify them using topological data analysis. This allows us to identify materials with similar pore geometries, and to screen for materials that are similar to given top-performing structures. Using methane storage as a case study, we also show that materials can be divided into topologically distinct classes requiring different optimization strategies.

## Abstract (web summary)

Pore structure plays an important role in dictating gas storage performance for nanoporous materials. Here, Smit and colleagues develop a topological approach to quantify pore structure similarity, and exploit the resulting descriptor to screen for materials that possess structural similarities with top-performers.

Understanding Big Data is a challenge social and natural sciences share. The need to handle huge amounts of data, often generated by the steady increase of available computing power, has inspired rapid development in big-data science. In chemistry and material science, new research initiatives (for example, the materials genome initiative^1^,^2^) have led to the generation of large databases of materials for different applications.

We focus on nanoporous materials, such as zeolites^3^, metal organic frameworks (MOFs)^4^, zeolitic imidizolate frameworks (ZIFs)^5^ and porous polymer networks (PPNs)^6^. These materials are of interest in applications ranging from gas separation and storage, to catalysis. In each case one would like to tailor-make a material that is optimal for that particular application. The chemistry of these materials allows us to obtain an essentially unlimited number of new materials^7^,^8^,^9^,^10^,^11^. Indeed, in recent years the number of published synthesized nanoporous materials has grown exponentially^4^. Yet, this growth is exceeded by the number of predicted structures, giving us libraries of millions of potentially interesting new materials. This sheer abundance of structures requires novel techniques from big data research to shed light on the existing libraries, as well as to facilitate the search for materials with optimal properties.

In nanoporous materials the shape of the pores plays an essential role in the behaviour of the material^12^,^13^. Conventionally, pore structure is characterized by a set of traditional geometric descriptors such as pore volume, largest included sphere, surface area and so on. These descriptors can be successfully optimized to search for materials with similar overall thermodynamic properties, but, as we will show, they capture partial geometric features only and do not encode enough geometric information to enable detection of materials that have similar overall pore shapes. There are computational techniques to quantify the similarity between crystal structures^14^,^15^. However, these algorithms are limited to identifying identical crystal structures, while we are interested in finding materials that may have different crystal structures or chemical compositions but similar pore geometries. Martin *et al*.^16^ developed Voronoi network representations of pore geometries, which are useful as fingerprints but do not capture details of the local pore structure.

We develop a mathematical quantification of geometric similarity by using topological data analysis (TDA). TDA is a field of big data analysis that builds on techniques from algebraic topology, most noticeably persistent homology^17^. Its guiding philosophy is that the ‘shape' of the data reveals important information about the data^18^.

## Results

### Developing a topological descriptor for pore shapes

To assign a geometric descriptor to a given material, we sample points on the pore surface. By growing balls stepwise around each sample point and monitoring their pairwise overlaps, we compute the associated filtered Vietoris-Rips complex, which is then characterized by its zero- (0D), one- (1D) and two-dimensional (2D) homology classes (see Supplementary Note 1 on mathematical background; Supplementary Fig. 3 for the homology classes of the torus and Supplementary Fig. 5 for the construction of the filtered Vietoris-Rips complex). We store the lifetime of each homology class in the corresponding persistence barcode. Combining the 0D, 1D and 2D barcodes yields a fingerprint that characterizes the overall shape of the pore structure.

**Figure 1:**
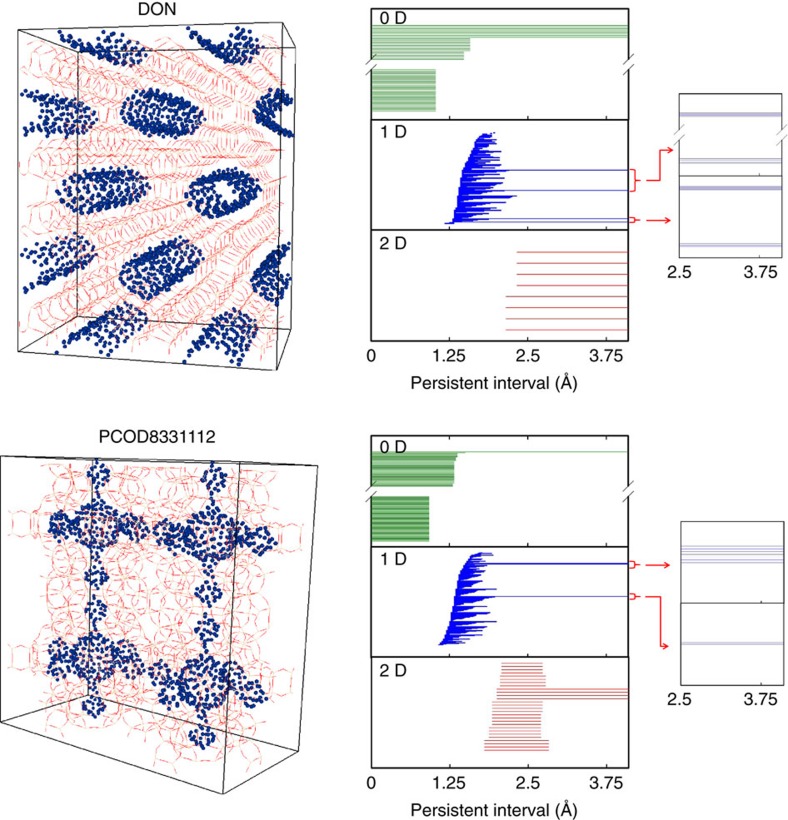
Examples of two zeolite fingerprints. The persistence barcodes of two different zeolite structures DON (top) and PCOD8331112 (bottom). The figures on the left show the structures, the middle the fingerprints and the right magnifies details of the 1D fingerprints. The red lines in the figures on the left show the zeolite structures, and the navy dots are the set of randomly sampled points on the pore surfaces. The Supplementary Movies 1 and 2 contain animations of growing these fingerprints.

For analysing pore shapes we are in the unusually fortunate situation that, unlike most other big-data applications of persistent homology, our data have actual geometric meaning. In almost all known big-data applications only the 0D and 1D barcodes are of relevance, while here the 2D barcodes also carry essential information. For example, Fig. 1 shows the fingerprints of two different zeolite structures, IZA zeolite DON and hypothetical zeolite PCOD8331112. DON contains eight identical cylindrical pores that run parallel to each other. The pore structure of PCOD8331112 is a three-dimensional (3D) network that is formed of two types of connected spherical cavities. The 0D barcodes of both structures start with as many intervals as there are points sampled on the pore surfaces. More information is contained in the long intervals describing robust shape features: the existence of the single long interval in its 0D barcode implies that the pore system of PCOD8331112 is connected. In contrast, the pore system of DON consists of eight disjoint components, encoded by the eight long intervals in its 0D barcode. The 1D and 2D barcodes contain information on the shape of the cavities (see Supplementary Note 1).

### Identifying structures with similar pore shapes

**Figure 2:**
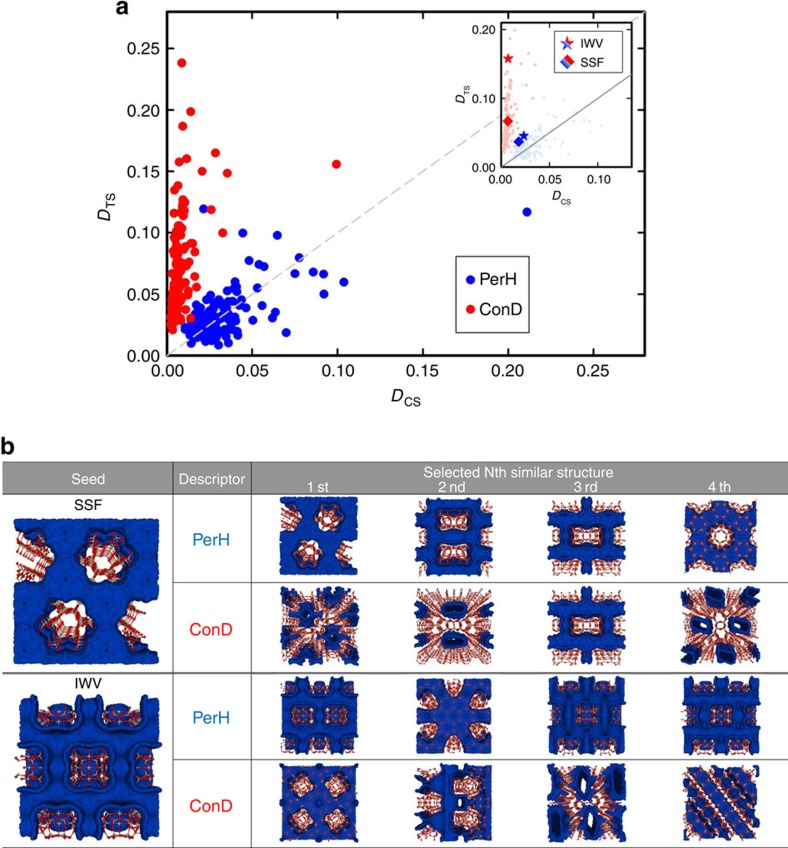
Structures similar to a reference material. (**a**) For each known zeolite, the two sets of four most similar structures, once selected using the TDA descriptor (PerH, one blue dot for a set of four) and once selected by the conventional descriptor (ConD, one red dot per set) are compared. This is done by plotting their average distances *D*_CS_ in conventional space (x-axis) and their average distance *D*_TS_ in the barcode space (*y* axis) to the reference zeolite. The distances are normalized by the largest pairwise distance in the database. (**b**) The four structures most similar to the zeolite SSF respectively to IWV, as selected by either PerH or ConD. Their structural properties are given in Supplementary Table 1. The inset in **a** highlights the four sets of four structures shown in **b**.

The most elementary, but highly non-trivial, application of our approach is to identify porous materials with similar pore structures. As we have a database of over 3,000,000 nanoporous structures^19^, visual inspection is out of the question. Suppose we would like to know whether the library of hypothetical zeolites contains structures whose pore geometry is similar to a given material, for example, a synthesized zeolite. To see the effectiveness of our approach, it is instructive to take a structure and find the four structures that are most similar to the chosen one, selected once by conventional descriptors (ConD) and once using persistent homology (PerH). To compare these two sets, we compute their average distances to the reference material, measured by the metric *D*_CS_ of the conventional space as well as by the metric *D*_TS_ of the barcode space (see Methods section for details). Figure 2a shows the average distances of the two sets for each of the 146 experimentally known zeolite structures accessible to methane. The distances are normalized by the largest pairwise distance in the database. The TDA approach provides what one would expect: when persistent homology is used to identify similar pore structures, small *D*_TS_ correlates well with small *D*_CS_, that is, similar persistence diagrams describing the pore shapes correlate with similar conventional geometric measures. Figure 2a shows that the relatively few zeolites for which there are no four structures very similar to a given one with respect to PerH (large *D*_TS_), the first four structures chosen by PerH might or might not have similar conventional geometric descriptors (small or large *D*_CS_). The conventional approach, however, gives a different result: for each reference structure we can find structures with similar conventional descriptors (small *D*_CS_) but the shapes of their pores can differ enormously (large *D*_TS_). Figure 2b shows two cases where the conventional approach identifies structures with very similar conventional descriptors (Supplementary Table 1) but very different pore structures. In contrast, if we use our topology-based fingerprint, we indeed retrieve structures that look strikingly similar. In the Supplementary Note 2 we show that one can also use this similarity search to compare structures from different classes of nanoporous materials. These findings are guaranteed by a stability theorem that is a key result in persistent homology^20^: materials with similar shapes are described by similar barcodes.

For the traditional descriptors with geometric meaning, one expects to find correlations with information encoded in the persistent homological fingerprint (Supplementary Note 3). For example, the radius of the maximum included sphere is correlated with the 2D barcode, as the radius of a cavity determines the death time of an interval in the 2D barcode. Further geometric information, like the connectivity of the pore structure (0D) or the number of independent tunnels (1D), is also encoded in the persistence barcodes (Supplementary Table 3). Therefore, only the combination of the barcodes of all three dimensions captures the global geometric features of the pore shapes we are interested in.

**Figure 3:**
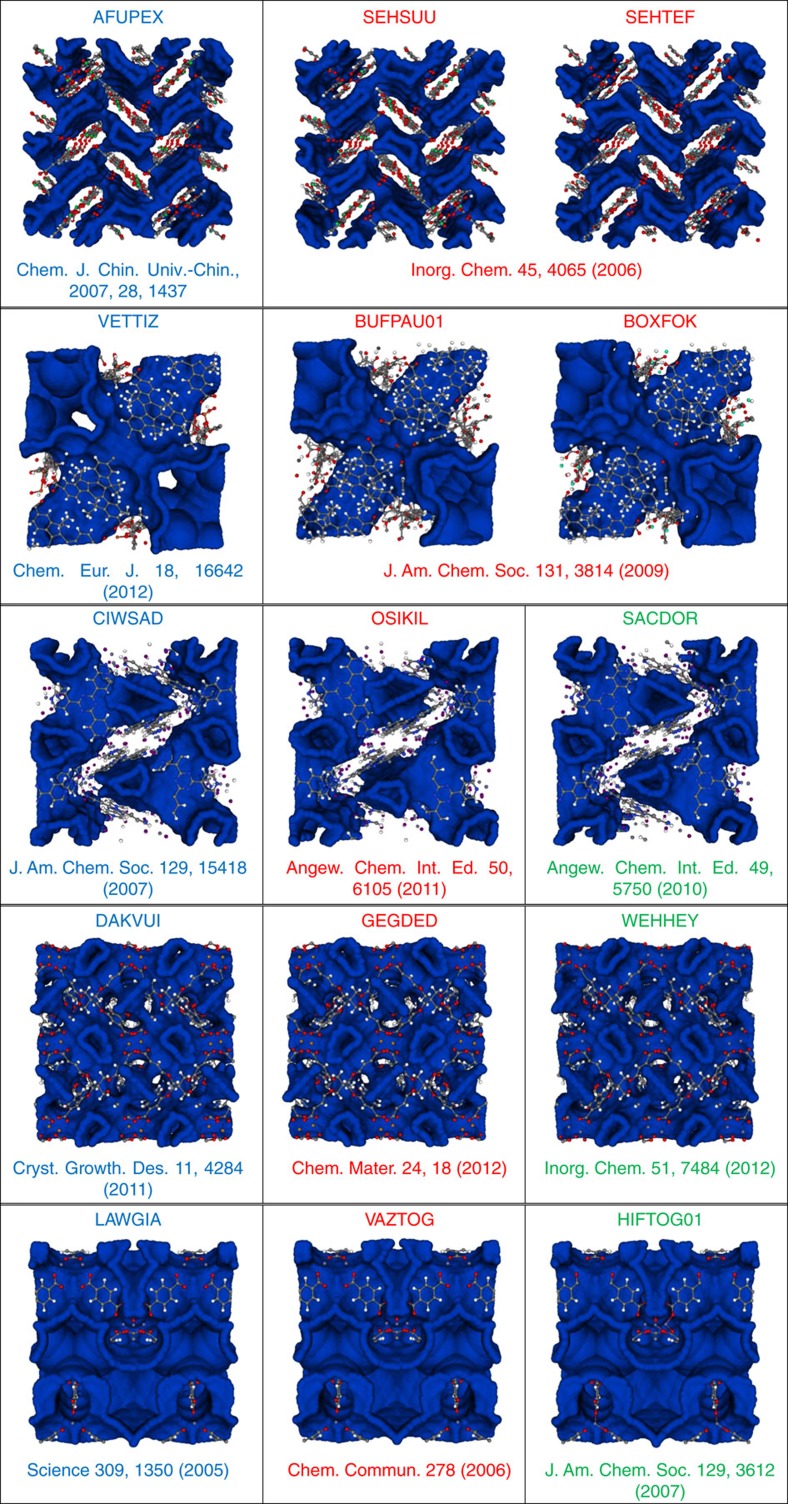
Materials from the CoRE-MOF database that have similar pore geometry. Each row gives examples of materials that are very similar. There are many more similar structures in the CoRE-MOF data base than we have listed here. The ones that are listed are those in which there are no cross references in the original articles of the corresponding similar structures.

One of the characteristics of MOFs is their chemical tunability. Indeed, over the last 5 years, over 10,000 structures have been synthesized^4^. Such a large number of materials makes it simply impossible to compare the corresponding pore structures visually. Therefore, an important application of our methodology is that we can now readily identify similar pore structures. In Fig. 3, we show some examples of materials from the CoRE-MOF database that have similar pore geometries. Our list of similar structures in much longer but what is specific to these examples is that the authors of the corresponding manuscripts did not report the similarities in the original references. Of course, this does not imply that the authors of these articles were not aware of these similarities, but given that there are over 10,000 experimental MOF structures, such similarities are easily overlooked.

### Methane storage case study

An important practical application of nanoporous materials is methane storage. The performance property of this application is deliverable capacity, which is defined as the difference between the amount of methane that is adsorbed at the (high) pressure at which the material is charged and the amount that remains in the material at the de-charging (low) pressure; the higher this deliverable capacity, the better the material. One of the interesting features of nanoporous materials is that one can optimize the pore geometry for a given application. The idea is that if one identifies a material with a high deliverable capacity, materials with similar pore geometries should also have an excellent performance.

**Figure 4:**
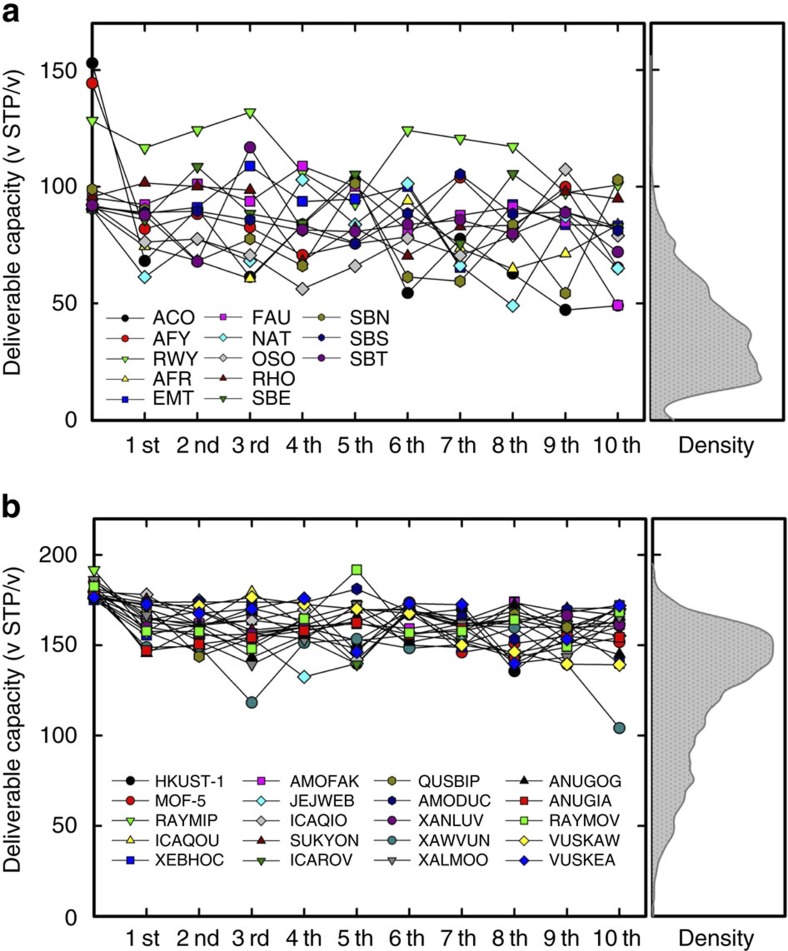
Deliverable capacity of materials similar to the known best performing materials. Deliverable capacity of the 10 materials that are most similar to the 13 best performing zeolites (**a**), respectively, 20 MOFs (**b**) with respect to PerH.

We illustrate this idea for all-silica zeolites. For this class of nanoporous materials the chemical composition (Si/O) is the same, hence the determining factor is the pore shape. From molecular simulations we have determined the 13 best performing out of the 180 known zeolite structures, each having a deliverable capacity larger that 90 (v STP/v). We subsequently identified for each of these top-performing materials the 10 most similar structures in our database of 139,407 predicted zeolites. Figure 4a shows that indeed 80% of these 130 new structures have a deliverable capacity that is similar to the 13 top-performing known zeolites. In Fig. 4b, we show a similar result for MOFs, where we used the 20 top-performing structures from the CoRE-MOF database and identified similar structure in the databases of 41,498 predicted MOF structures: 85% of these materials show high performance with a deliverable capacity larger than 150 (v STP/v). It is interesting that even for MOFs that have different chemical compositions (unlike zeolites), our method of identifying similar pore shapes illustrates the importance of pore geometry, and hence, of our methodology to quantify similarity for these types of materials.

**Figure 5:**
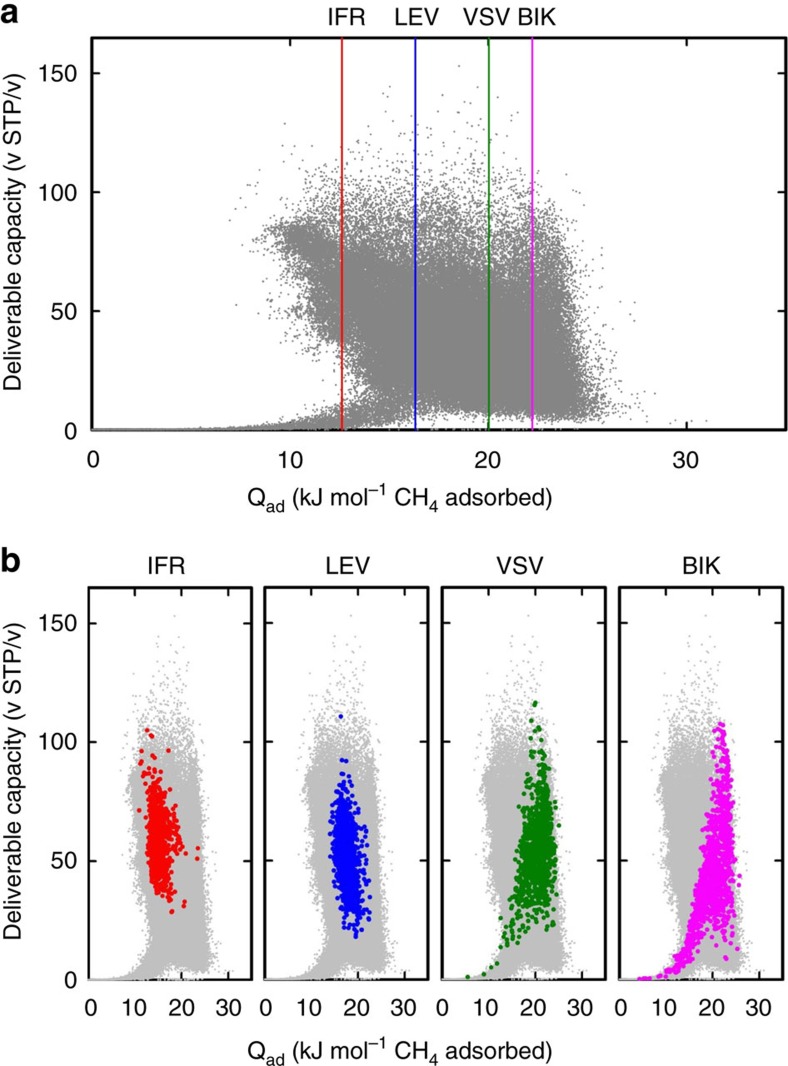
The deliverable capacity and heat of adsorption of zeolites. (**a**) The deliverable capacity and heat of adsorption of all zeolites (data from Simon *et al*.^12^). (**b**) Four reference structures IFR, LEV, VSV and BIK were chosen. For each of them we show the 500 geometrically most similar materials (with respect to our topological descriptor) highlighted on the plot from **a**. The optimal heats of adsorptions for these subsets are depicted with the vertical lines in **a**.

We can also use our approach to study the topological diversity of the top-performing materials for methane storage. Bathia and Myers^21^ analysed a small number of porous materials and concluded that top-performing materials should all have very similar heats of adsorption for given loading and de-charging pressures. Their work has had significant impact, as it provides a straightforward experimental recipe for optimizing the deliverable capacity of a material^22^: if all top-performing materials share a similar heat of adsorption, having a heat of adsorption close to this value should be a necessary condition for good performance. Given this impact it is surprising that the conclusion of Bathia and Myers^21^ stands in sharp contrast with observations of Simon *et al*^12^. Simon *et al*.^12^ computed the deliverable capacity for over 200,000 zeolite structures, and their data (Fig. 5a) provide no evidence for a single optimal heat of adsorption, pointing to an interesting paradox: if one randomly selects a set of materials from Fig. 5a, one finds no experimental indication that an optimal heat of adsorption even exists. Yet, the approach of Bathia and Myers has indeed been shown to be useful in optimizing performance.

To shed some light on this paradox, we applied topological data analysis to the data in Fig. 5a. Analysing the heat of adsorption for sets of geometrically similar structures, we obtain the desired ‘volcano plots' shown in Fig. 5b, which allow us to systematically search for the optimal heat of adsorption within a class of geometrically similar structures, and hence the best-performing materials. Interestingly, this optimal heat of adsorption depends on the geometric type of a material^23^,^24^ (Fig. 5) and is not, as suggested by Bathia and Myers, a universal constant. In fact, Bathia and Myers assume implicitly that the entropy of adsorption is the same for all materials; for a set of similar materials as often chosen this assumption is more likely to hold.

**Figure 6:**
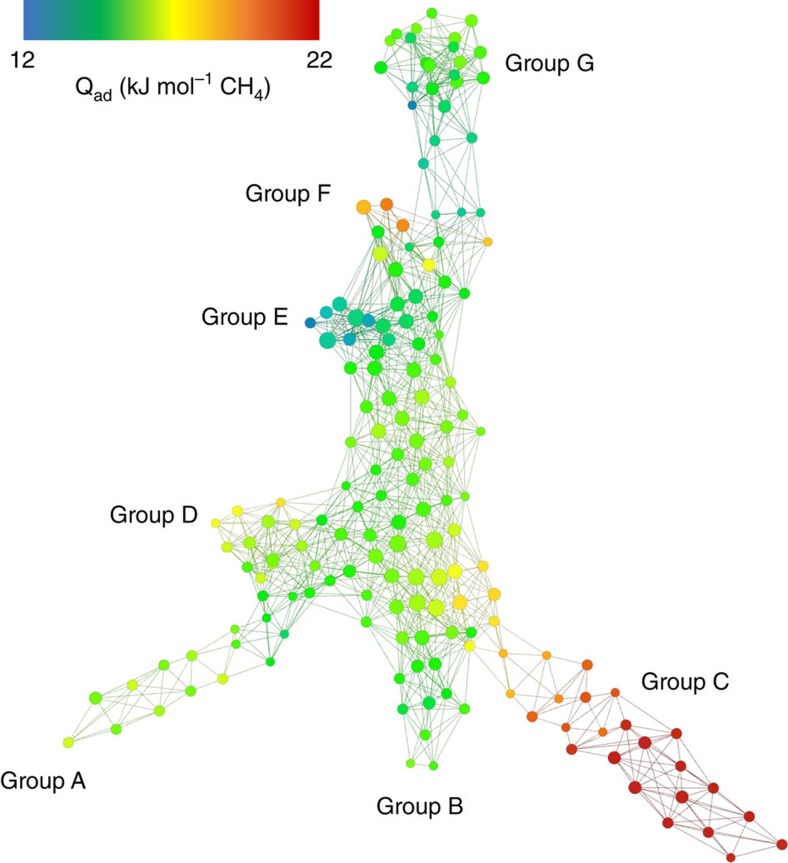
Mapper plot of the best zeolites (top 1%) for methane storage. Nodes in the network represent clusters of materials with similar pore shapes, and edges connect nodes that contain structures in common. Each material is represented by a combination of its persistence barcodes, and the metric in this space is *D*_TS_. Examples of materials from the different groups are shown in Supplementary Table 2. These figures were obtained with the Ayasdi Core software platform (www.ayasdi.com). Nodes are coloured by the average value of the heats of adsorption of the materials in a cluster (Red: high value, Blue: low value).

The results above suggest that there is not a single class of optimal materials. For this particular example Simon *et al*.^12^ carried out brute force simulations to compute the performance of all materials. We could therefore apply TDA to analyse the geometric diversity of the top-performing structures and to visualize the topography of the zeolite library by generating the mapper plot^18^,^25^ shown in Fig. 6, encoding the topological structure of the set of the top 1% of zeolites with respect to methane storage.

The shape of the diagram shows seven topologically different classes of top-performing materials. For example, group C consists of materials that have one-dimensional small cylinders, while group E has two-dimensional channels (see Supplementary Table 2 for all different groups). The colour coding of the mapper plot nicely illustrates that materials in classes of different pore shapes have very different optimal heats of adsorption.

## Discussion

Using topological data analysis, we have developed a topology-based methodology to quantify similarity of the chemical environment of adsorbed molecules. Quantifying similarity of pore structures allows us not only to find structures geometrically similar to top-performing ones, but also to organize the set of materials with respect to the similarity of their pore shapes. For our case study of methane storage, we find several distinct classes of pore shapes and conclude that each class requires a different optimization strategy, in contrast to the common belief that top-performing materials share a similar heat of adsorption. We give examples of geometrically almost indistinguishable MOFs whose similarities had previously been unreported (Fig. 3). The Supplementary Information shows the hypothetical zeolites that best resemble MOF-5 and CU-BTC (Supplementary Fig. 6), and contains examples of hypothetical MOFs that are similar to synthesized MOFs (Supplementary Fig. 7).

In this work, we focus on applications in which the pores play a passive role in providing adsorption sites. For applications in which the pores play a more active role, such as catalysis, a logical step would be to extend the methodology to include chemical specificity and charge distribution. From a topology viewpoint this application is of particular significance because it is one of the first applications of topological data analysis that requires persistent homology in three different dimensions.

## Methods

### Generating the barcodes

To assign the persistent homological descriptor to a material, we perform the following steps. We start by preparing a supercell of the material by expanding each unit cell to approximately the size of the largest unit cell of all considered materials, to compare materials that have unit cells of very different sizes. The pore system accessible to a gas molecule of interest is determined using the software package Zeo++ (ref. 16). The surface of this pore system is sampled with a fixed number of points per unit surface area. From these sampled points, filtered Vietoris-Rips complexes are constructed and their 0D, 1D and 2D persistence barcodes computed using the software package Perseus^26^. We measure the distance between two barcodes by a combination of the *L*^2^-landscape distances of the barcodes from the dimensions 0,1 and 2 (see section below), using the Persistence Landscape Toolbox^27^.

The program Zeo++ (ref. 16) detects the accessible void space inside a porous material using a periodic Voronoi network, modelling the framework atoms as hard spheres with radii taken from the Cambridge Structural Database^28^,^29^. The space accessible to a gas depends on the gas molecule size and is determined in terms of a probe gas molecule, where the size of the probe has to be chosen according to the specific application. We treat a probe gas molecule as a sphere with radius 1.625, 1.5, 1.83 or 1.98 Å for methane, carbon dioxide, krypton or xenon, respectively. These values are chosen smaller than usual to mimic by geometric constraints the accessibility of pore space as determined by energy barriers. Zeo++ encodes the pore structure as a large set of points situated on the pore surface which is defined as the boundary of the space where a probe can be placed. For example, a cylinder-shaped pore whose radius equals the probe radius will be represented by points along the central line of the pore.

To analyse this point cloud with persistent homology tools, it is necessary to decrease the number of points by performing a secondary sampling, since the raw output is too large to be handled: hundreds of thousands of points for each supercell. On the one hand, it is important to have a fine enough resolution to capture details of the pore structures using only finitely many points and to ensure that the barcode assignment is stable with respect to the choice of the point cloud. On the other hand, high resolutions increase computational costs for the persistence computation. We use a combination of random sampling and grid sampling. The grid sampling guarantees that different samplings of a structure give comparable barcodes, in particular by ensuring that points on narrow parts of the pore system are sampled while still maintaining its connectivity. On the other hand, the random sampling prevents picking up the grid structure in the barcodes. For the random sampling we choose one point per 2 Å^2^ surface area while respecting a minimal distance *r*_min_ between two sampled points where we decrease *r*_min_ in steps of 0.1 Å starting with *r*_min_=1.3 Å until the given number of points has been selected. The grid size is 0.5 Å and for each cube of the grid the point of the original point cloud is chosen that is closest to the midpoint of the cube. A point of the grid sampling is added to the random sampling whenever its distance to the randomly sampled points is greater than the final value of *r*_min_.

The second step towards the persistent homological descriptor consists of calculating the persistence barcodes for a filtration of Vietoris-Rips complexes, obtained from the sets of points computed in the first step using the software package Perseus^26^. We restrict ourselves to constructing 3D Vietoris-Rips complexes, where the filtration parameter 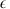 (corresponding to the radius of the balls grown around each point) increases in 164 steps of 0.025 Å increments, starting from the initial value of 0. The resulting 4.1 Å maximal filtration parameter is due to the fact that the memory cost of using Perseus grows extremely fast as the radius increases in our calculations. While the relatively small maximal filtration parameter does not allow us to build a complete complex, it prevents geodesically distant points of the surface that are close in Euclidean metric to be connected unless the pore structure is very densely packed in the material. This is important since our construction does not distinguish homology classes that are formed in the solid part of the material from those formed in the pore regions. Technically, this makes the descriptor an overall descriptor of the geometry of the embedding of the pore-surface in the ambient space and not strictly describing the pore surface with respect to the pore space only. Fortunately, the technique does not tend to misidentify structures since the material part is typically much larger than the pore part. However, our maximal filtration parameter is not sufficiently large for all homology classes to die—these correspond to essential intervals in the barcodes—especially for zeolites having large pores. Therefore, to take account of these homology classes in computing distances between two barcodes, a maximal value for the death time has to be assigned, which is especially important in dimension 2 because of the small cardinality of barcodes. For 2D barcodes, we assign a death times to essential intervals based on the relation between the diameter *D*_i_ of the largest included sphere and the death time for small and medium pores which is linearly fitted. An example for zeolites with methane is shown in Supplementary Fig. 1. The 1D barcodes contain sufficiently many intervals to distinguish different structures, and we discard essential intervals.

### The metric of the barcode space

To quantify the similarity between two materials in the barcode space, we combine as follows the *L*^2^-distances between the persistence landcapes (see Supplementary Note 1, and Supplementary Fig. 4) corresponding to their barcodes in the different dimensions. After testing landscape distances of different orders (that is, *L*^∞^, *L*^0^, *L*^1^, *L*^2^ and so on), *L*^2^-distances were chosen because they gave the smallest errors in predicting global structural properties and performance properties for a test set of materials. Let Λ_*d*=1_ (respectively, Λ_*d*=2_) be the *L*^2^-landscape distance between the 1D (respectively, 2D) persistence barcodes, and let *L*_0_=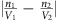 where *n*_*i*_ is the number of points sampled on the pore surface of the *i*th material, and *V*_*i*_ is the volume of the supercell. The distance between two materials in the barcode space is then


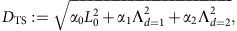


with coefficients *α*_0_=0.1, *α*_1_=0.45 and *α*_2_=0.45, the values of which were chosen to minimize the error in predicting global structural properties and performance properties for a test set of materials. In dimension 0 the essential intervals are effectively discarded, and instead of the 0D barcode, the number of sampled points per unit volume is used. This is a simplification that corresponds to discarding the essential intervals in all cases where different connected components of the pore system stay separated during the entire filtration; the 0D barcodes of connected components are determined by the sampling procedure by construction.

The distance *D*_CS_ between two materials in the conventional descriptor space is estimated with a normalized euclidean distance of five conventional structural properties with an equal weight for each: *D*_i_ (the diameter of largest included sphere), *D*_f_ (the diameter of largest free sphere), *ρ* (density of a framework), ASA (accessible surface area), and AV (accessible volume). The dependence on the choice of the weights is shown in Supplementary Fig. 2.

### Mapper plot

We used Ayasdi 3.0 Core software (www.ayasdi.com) to visualize our materials database (Fig. 6). Nodes in the network represent clusters of materials with similar pore shapes and edges connect nodes that contain structures in common. Each material is represented by a combination of its persistent barcodes and the metric in this space is *D*_TS_. The lens used to bin the barcodes is the neighborhood lens (resolution 30, gain 3.0 × ). Further information can be found in the Ayasdi manual. Nodes are coloured by the average value of the heats of adsorption of the materials in a cluster (Red: high value, Blue: low value).

### Data availability

Barcodes that support the findings of this study are available in ‘Materials Cloud', ‘ http://materialscloud.org/archive/2017.0001/v1/'. Access to any of other data sets can be requested by writing to the corresponding author.

## Additional information

**How to cite this article:** Lee, Y. *et al*. Quantifying similarity of pore-geometry in nanoporous materials. *Nat. Commun.* **8,** 15396 doi: 10.1038/ncomms15396 (2017).

**Publisher's note**: Springer Nature remains neutral with regard to jurisdictional claims in published maps and institutional affiliations.

## Supplementary Material

Supplementary Information Supplementary Figures, Supplementary Tables, Supplementary Notes and Supplementary References

Supplementary Movie 1 Fingerprints for a IZA zeolite DON. The upper part shows the procedures to identify pore structures inside a IZA zeolite DON and perform persistent homology analysis for sampled points (navy dots) on pore surfaces. The bottom part shows evolution of barcodes over persistent interval.

Supplementary Movie 2 Fingerprints for a hypothetical zeolite PCOD8331112. The upper part shows the procedures to identify pore structures inside PCOD8331112 and perform persistent homology analysis for sampled points (navy dots) on pore surfaces. The bottom part shows evolution of barcodes over persistent interval.
