# Supplementary material for: Quantifying similarity of pore-geometry in nanoporous materials
Source: Nat Commun. 2017 May 23;8:15396. doi: 10.1038/ncomms15396 (PMC5457500; doi:10.1038/ncomms15396)
Supplement: Supplementary Information — Supplementary Figures, Supplementary Tables, Supplementary Notes and Supplementary References [file ncomms15396-s1.pdf]

Supplementary Figures

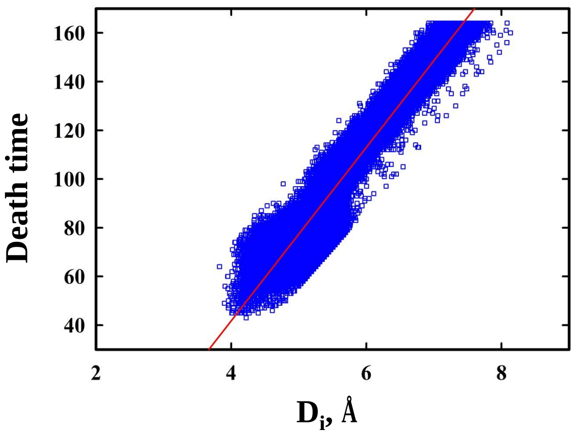

Supplementary Figure 1: Correlation of the death time of 2-dimensional homology classes and the diameters of the largest included sphere D_i when using methane CH_4 as a probe molecule. The red line indicates the least squares regression line; Death time = $35.6 \times D_i - 100.8$.

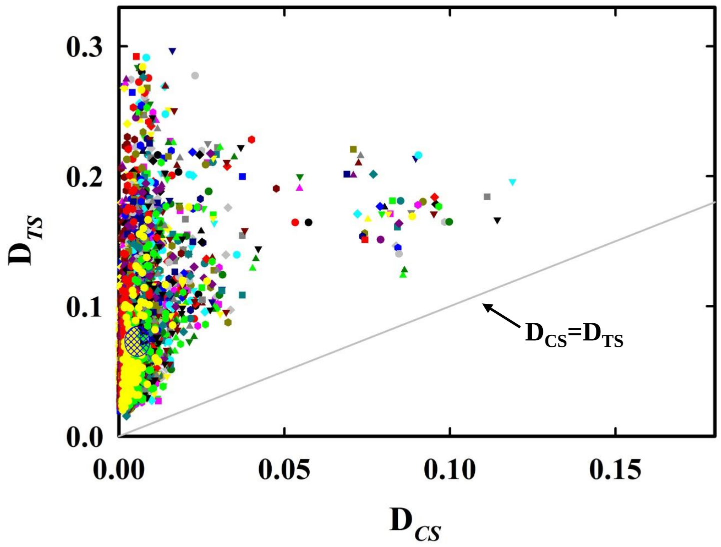

Supplementary Figure 2: The effect of weighting the conventional properties differently on ConD. The x and y axes give the average of the distances (D_{CS} or D_{TS}) from four most similar materials to the corresponding reference zeolite structure (seed structure for searching similar ones) measured in conventional or barcode space respectively. 50 different combinations of weight factors were chosen randomly, and the results for each combination are distinguished using different colors. A cross-hatched ellipse shows the area that contains the centers of the point clouds which are obtained from different weighting choices.

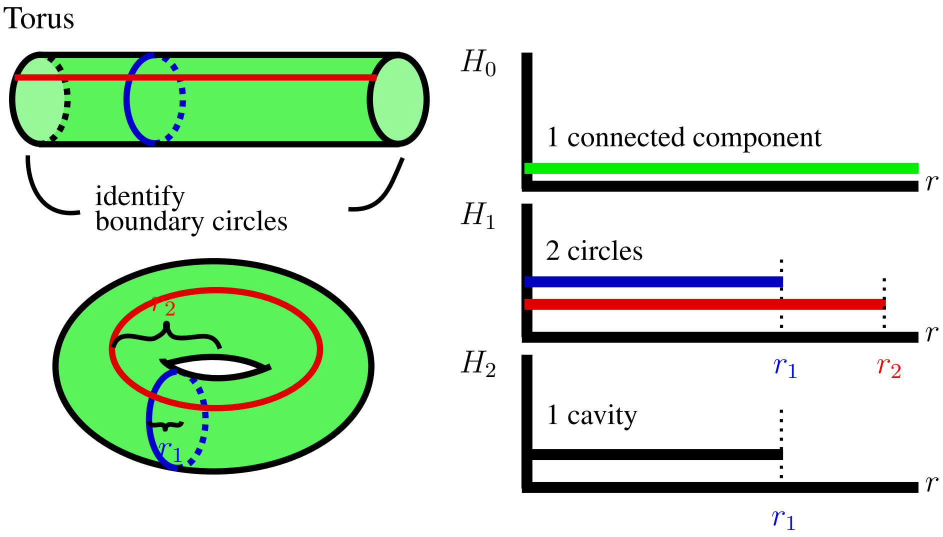

Supplementary Figure 3: The persistence barcodes of a torus as obtained from a channel by implying periodic boundary conditions.

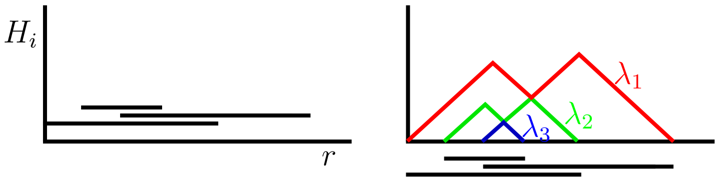

Supplementary Figure 4: A persistence barcode and its corresponding persistence landscape.

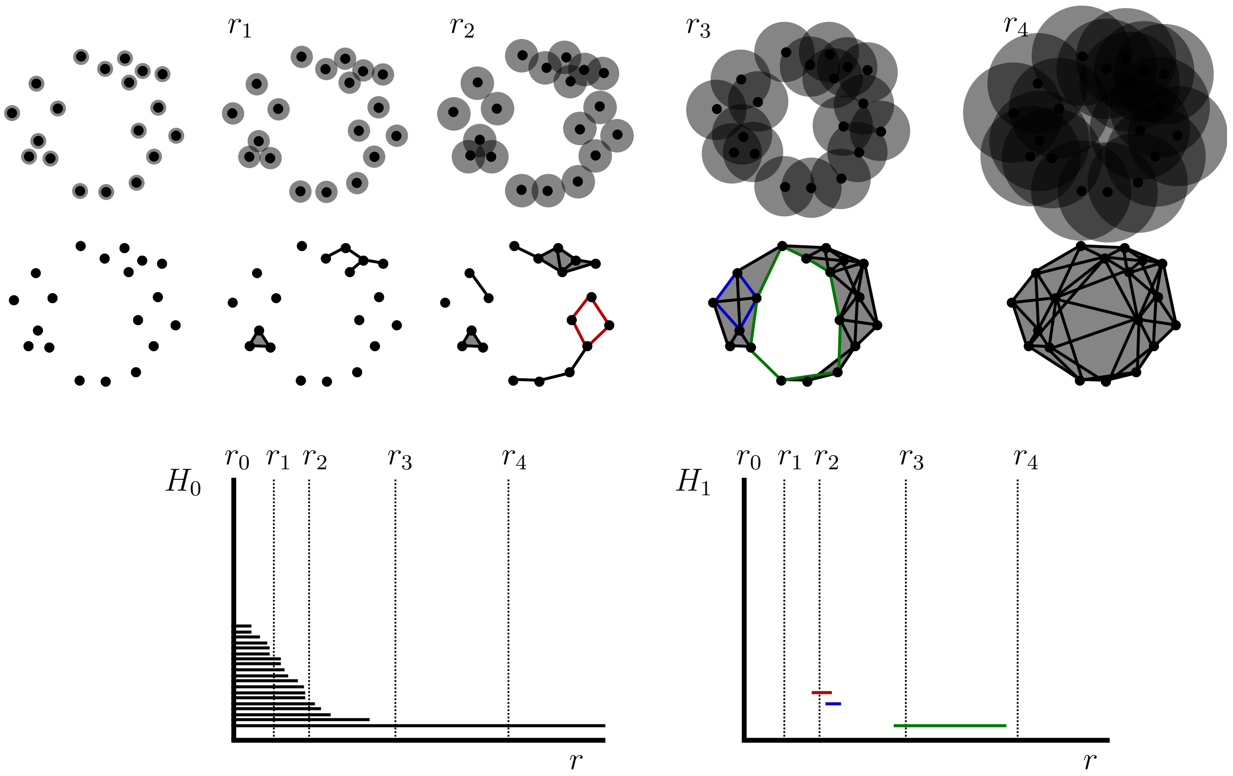

Supplementary Figure 5: Construction of the Vietoris-Rips complex from a point cloud in 2D for increasing radii, together with the 0- and 1-dimensional persistence barcodes of the resulting filtration. H_0 counts the connected components of the complex for a given radius, and H_1 tracks circles that do not bound disks. The construction in 3D works analogously, using balls instead of disks.

Seed	TD
MOF-5 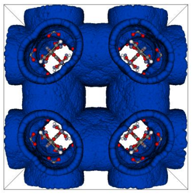	PCOD8307680 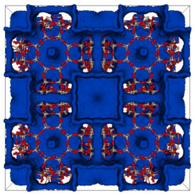
Cu-BTC 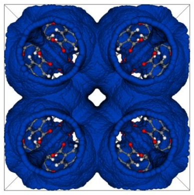	PCOD8162096 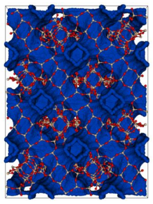

Supplementary Figure 6: The zeolites most similar to MOF-5 and Cu-BTC with respect to PerH.

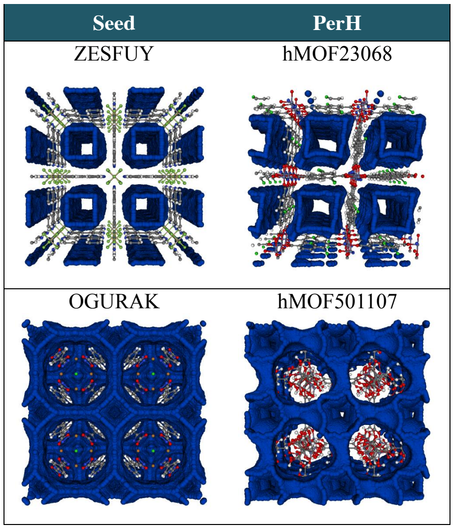

Supplementary Figure 7: Finding hypothetical MOFs that best resemble the experimentally known structures ZESFUY and OGURAK.

Supplementary Tables

Descriptor		name	D_i	D_f	ρ	ASA	AV
	Seed	SSF	7.59	6.15	1.64	1191.97	0.122
	1st	PCOD8328603	8.09	6.34	1.77	1167.86	0.120
PerH	2nd	PCOD8267032	7.54	6.22	1.70	1171.01	0.115
	3rd	PCOD8267258	7.72	6.21	1.63	1205.27	0.115
	4th	PCOD8325065	7.63	6.29	1.59	1239.91	0.133
	Seed	SSF	7.59	6.15	1.64	1191.97	0.122
	1st	PCOD8242590	7.87	6.16	1.62	1210.05	0.119
ConD	2nd	PCOD8239380	7.60	6.29	1.63	1156.76	0.120
	3rd	PCOD8267258	7.72	6.21	1.63	1205.27	0.115
	4th	PCOD8070132	7.69	6.49	1.62	1187.66	0.126
	Seed	IWV	8.48	6.97	1.50	1502.63	0.181
	1st	PCOD8285528	8.46	6.86	1.54	1476.78	0.176
PerH	2nd	PCOD8329417	7.88	6.65	1.49	1507.05	0.189
	3rd	PCOD8284133	9.17	6.92	1.63	1491.56	0.194
	4th	PCOD8283909	9.08	6.60	1.49	1534.93	0.187
	Seed	IWV	8.48	6.97	1.50	1502.63	0.181
	1st	PCOD8302674	8.78	7.00	1.50	1499.77	0.183
ConD	2nd	PCOD8310713	8.39	7.05	1.49	1533.70	0.178
	3rd	PCOD8079814	8.33	7.07	1.48	1523.32	0.175
	4th	PCOD8059487	7.99	7.01	1.48	1506.13	0.180

Supplementary Table 1: The global structural properties of the four zeolites most similar to SSF and IWV selected by either conventional descriptors (ConD) or using persistance homology (PerH): D_i (maximum included sphere), D_f (maximum free sphere), ρ (density), ASA (accessible surface area), and AV (accessible volume).

	Examples			Features
Group A	PCOD8195264 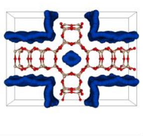	PCOD8196363 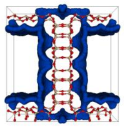	PCOD8196357 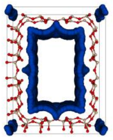	• 1-dimensional channels<br>• Large equilateral polygonal cross-section
Group B	PCOD8269881 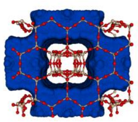	PCOD8283782 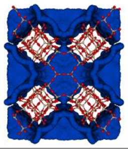	PCOD8312127 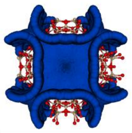	• 1-dimensional channels with connections
Group C	PCOD8053175 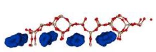	PCOD8053791 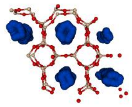	PCOD8101338 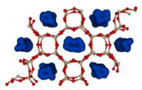	• 1-dimensional channels<br>• Small cross-section<br>• Multi channels
Group D	PCOD8195610 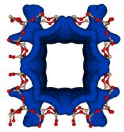	PCOD8274956 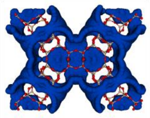	PCOD8312656 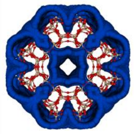	• 2-dimensional channels
Group E	PCOD8193734 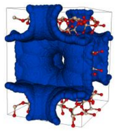	EMT 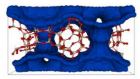	FAU 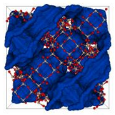	• Connected small polygonal cross-section<br>• Other shapes
Group F	PCOD8123217 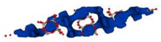	PCOD8106667 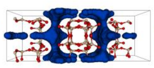	PCOD8202177 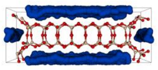	• Flatten channels<br>• Small void fraction
Group G	PCOD8078190 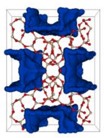	PCOD8059318 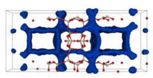	PCOD8235214 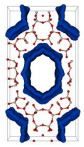	• Small polygonal cross-section without connection

Supplementary Table 2: Examples from the seven topologically different classes of top-performing materials for methane storage (see also Figure 4).

Property	PD_0	PD_1	PD_2	$PD_{1,2}$	$PD_{0,1,2}$
K_H^*	0.080	0.087	0.074	0.085	0.086
ρ	0.082	0.107	0.121	0.096	0.073
Q_{ad}	0.369	0.392	0.412	0.379	0.386
ASA	0.078	0.476	0.621	0.459	0.091
D_i	0.318	0.367	0.155	0.234	0.172
D_f	0.346	0.263	0.344	0.293	0.158
AV^*	0.217	0.328	0.319	0.312	0.194

*Mean absolute percentage errors of K_H and AV are obtained with $\log K_H$ and $\log AV$.

Supplementary Table 3: TDA analysis of the conventional descriptors K_H (Henry coefficient), Q_{ad} (heat of adsorption), ρ (density), D_i (maximum included sphere), D_f (maximum free sphere), ASA (accessible surface area), and AV (accessible volume). The data show the mean absolute percentage error, expressing how well these descriptors can be predicted on the basis of a training set using only the 0-D, 1-D or 2-D barcode as fingerprint, (PD_0, PD_1, and PD_2, respectively), the combined 1-D and 2-D barcodes ($PD_{1,2}$), and the combination of barcodes from all 3 dimensions ($PD_{0,1,2}$). The mean absolute percentage error is calculated as $\frac{1}{n}\sum_{i=1}^{n}\left|\frac{P_{i,PD}-P_i}{P_i}\right|$ where n is the number of zeolites, and P_i or P_i, $P_{i,PerH}$ is a property of a zeolite or that of the most similar zeolite selected by PD, respectively.

Supplementary Note 1

Persistent homology

Topological data analysis (TDA) is a mathematical technique for assigning various topological invariants to data. The guiding philosophy of TDA is that the 'shape' of the data, encoded by a mathematical 'signature', should reveal important relations among the data points. One of the best-known TDA techniques is persistent homology[1,2], which we describe very briefly here.

Each material is encoded as a point cloud obtained by sampling points on the pore surface, giving rise to a description of the material in terms of the coordinates of the sampled points in 3-space. From the points, we construct a filtration of Vietoris-Rips complexes, which is a sequence of nested triangulated objects.

For a fixed non-negative real number r, the Vietoris-Rips complex of a point cloud is constructed as follows from the collection of balls of radius r centered at the points of the point cloud. Starting with the elements of the point cloud, add a line segment between a pair of points when the balls centered at the two points overlap. Similarly, a solid triangle is added when each pair of balls centered at its corners intersect and a solid tetrahedron when four balls all intersect pairwise. This procedure can be extended to all higher dimensions, but we stopped at solid tetrahedra both for computational reasons and because our point cloud represented a real three-dimensional structure. Since the Vietoris-Rips complex for a small radius is contained in the complex for a bigger radius, we obtain a filtration of complexes (Supplementary Figure 5 top).

The shape of a complex is partly captured by its homology groups H_n, where n is a non-negative integer. The nonzero elements of H_n are the homology classes in dimension n, which correspond to the n-dimensional 'holes' in the complex. More precisely, the 0-dimensional homology classes correspond to the connected components, while a 1-dimensional homology

class is represented by a closed curve that does not bound a surface and a 2-dimensional homology class by a bounded cavity.

For example, a hollow tube has one 0-dimensional homology class since it is connected, one 1-dimensional homology class corresponding to the circle running around the axis of the tube, which does not bound a disk, and no 2-dimensional homology class, as the tube does not bound a 3-dimensional cavity. In contrast, if the ends of the tube are glued together, for example by applying periodic boundary conditions, a torus is formed (see Supplementary Figure 3). Being connected, it still has one 0-dimensional homology class, but two independent 1-dimensional homology classes, which are represented by the circle around the axis (blue) and the newly formed circle that runs parallel to the axis (red). The torus is hollow and thus bounds a cavity that represents a 2-dimensional homology class. If a solid torus is considered, the circle around the axis bounds a disk and therefore does not contribute to the 1-dimensional homology, and there is no nonzero 2-dimensional homology class.

The homology classes of a point cloud (such as that obtained by sampling a pore surface) are not very informative, since each point forms its own connected component, while $H_n = 0$ for all $n > 0$. In contrast, the homology groups of its Vietoris-Rips complexes strongly depend on the position of the points in space. This information is stored in persistence barcodes that track the non-trivial homology classes through the radius-dependent filtration. A persistence barcode is a set of intervals where each nontrivial homology class is represented by a bar. The starting point of an interval denotes the smallest radius for which the homology class represented by the interval (e.g., a circle around a hole in dimension 1) appears in homology of the associated Vietoris-Rips complex, while the endpoint is given by the radius where the homology class disappears (e.g., the smallest radius for which the balls close the hole) (Supplementary Figure 5 bottom, Supplementary Figure 3). Classes that have a short lifetime can be considered as noise, while classes that persist through long intervals reveal actual structural features of the point

cloud.

To compare two materials in terms of their persistence barcodes, we use a combination of the L^2-distances between the persistence landcapes corresponding to the persistence barcodes of same dimensions. Informally, a persistence landscape is a family of functions

$$\lambda = \left\{ \lambda_k : \mathbb{R} \to \mathbb{R} \cup \{\infty\} \mid k \in \mathbb{N} \right\}$$

obtained from a barcode by "stacking together isosceles triangles whose bases are the intervals of the barcode," where the kth function describes the contour of the kth maximum (Supplementary Figure 4); see **Supp Ref** 3 for a rigorous definition. The L^2-landscape distance between two persistence barcodes B and B' with corresponding persistence landscapes λ and λ' is given by

$$\Lambda(B, B') = \|\lambda - \lambda'\|_2 = \sum_{k=1}^{\infty} \left(\int |\lambda_k(t) - \lambda'_k(t)|^2 dt \right)^{\frac{1}{2}}$$

Supplementary Note 2

Similarity in different classes of nanoporous materials

To illustrate the application of our method to finding similar pore geometries across different classes of nanoporous materials, we consider the following questions.

1. Are there zeolites that have the same pore geometry as a given MOF?

2. Are there hypothetical MOFs that are similar to MOFs that have already been synthesized?

The common theme behind these questions is to illustrate how the methodology developed here allows researchers to identify materials that have similar pore geometries.

MOFs and zeolites

In Supplementary Figure 6, we identify the structures in the IZA+ hypothetical zeolite database[4,5] that are most topologically similar to some of the best known MOFs (e.g., MOF-5 and Cu-BTC). The figure shows that we can indeed find hypothetical zeolite structures that look very similar to these MOFs.

Hypothetical and experimental MOFs

For hypothetical MOFs we have a database of over 140,000 materials.[6] An interesting question is whether pore geometries similar to those occurring in hypothetical MOFs have already been synthesized. This question is difficult to answer with traditional methods, since the materials might differ in their chemical composition. We have compared the similarity of structures from the database of hypothetical MOFs (hMOFs) with the experimental structures in the CoRE-MOF database. Supplementary Figure 7 shows two examples of similar structures. The color

coding of the structures shows that the chemical composition of the two structures is very different.

Supplementary Note 3

Global structural properties

In the main text we explained that the different dimensions of the persistent homology of the structures that we consider admit geometric interpretation. It is therefore interesting to see whether we can detect this geometric content when we use our method to test the capability of PerH to screen zeolites for the following conventional structural properties: D_i, D_f, ρ, ASA, AV, the Henry coefficient (K_H), and the heat of adsorption (Q_{ad}). We use methane as a probe molecule.

Starting with a highly diverse training set of 600 structures chosen by the min-max algorithm[7], we perform high-throughput screening for the entire set of zeolites, using five different PerH's: PD_0 (=L_0 as defined in Methods), PD_1, and PD_2, which use only 0-, 1-, or 2-dimensional persistent homology information respectively, as well as PD_{12} and PD_{012} which combine information from 1- and 2- or 0-, 1-, and 2-dimensional persistent homology. For PD_{12} equal weights were used, and for PD_{012} the same weights as given in the methods section. For each screening, we compare the conventional properties of each zeolite with those of the most similar one in the training set. Supplementary Table 3 summarizes the mean absolute percentage errors (MAPE) for each property, which is calculated as

$$\text{MAPE} = \frac{1}{n}\sum_{i=1}^{n}\left|\frac{PP_{i,PerH} - PP_i}{PP_i}\right|,$$

where n is the number of zeolites in the promising set, and PP_i (respectively, $PP_{i,PerH}$) is the performance property of the ith zeolite (respectively, of the zeolite in the training set most similar to the ith zeolite).

We observe that TDA-based descriptors are also capable of screening for structural properties. Moreover, the different dimensions of the persistent homology detect different structural properties. Using the standard deviation of the prediction of the screening as a measure of the

quality of the description, Supplementary Table 3 shows that the best prediction for the surface area (ASA) is the 0-dimensional descriptor (PD_0), while the maximum included sphere (D_i) is best predicted with a 2-dimensional descriptor (PD_2). In Supplementary Note 1, we explain that this corresponds to the geometric interpretation of the persistent homology in the different dimensions. In addition Supplementary Table 3 shows that averaging over the three dimensions provides a good description of all properties.

Supplementary References

1. Edelsbrunner H. and Harer J. L., *Computational Topology: an introduction* (American Mathematical society, Providence RI, 2010).

2. Carlsson G., Topology and data. *Cull Amer. Math. Soc.* **46,** 255–308 (2009).

3. Bubenik P., Statistical topological data analysis using persistence landscapes. *J. Mach. Learn. Res.* **16,** 77–102 (2015).

4. Pophale R., Cheeseman P. A. and Deem M. W., A database of new zeolite-like materials. *Phys. Chem. Chem. Phys.* **13,** 12407–12412 (2011).

5. Deem M. W., Pophale R., Cheeseman P. A. and Earl D. J., Computational discovery of new zeolite-like materials. *J. Phys. Chem. C.* **113**, 21353–21360 (2009).

6. Wilmer C. E., Leaf M., Lee C. Y., Farha O. K., Hauser B. G., Hupp J. T. and Snurr R. Q., Large-scale screening of hypothetical metalorganic frameworks. *Nat. Chem.* **4,** 83–89 (2012).

7. Kennard R. W. and Stone L. A., Computer aided design of experiments. *Technometrics* **11,** 137-148 (1996).
